# Comprehensive Biomarker Testing of Glycemia, Insulin Resistance, and Beta Cell Function Has Greater Sensitivity to Detect Diabetes Risk Than Fasting Glucose and HbA1c and Is Associated with Improved Glycemic Control in Clinical Practice

**DOI:** 10.1007/s12265-014-9577-1

**Published:** 2014-07-29

**Authors:** Stephen A. Varvel, Szilard Voros, Dawn L. Thiselton, James V. Pottala, Tara Dall, G. Russell Warnick, Joseph P. McConnell, Leila Ghaedi, Maciek Sasinowski, Timothy Graham

**Affiliations:** 1 Health Diagnostic Laboratory, Inc., 737, Richmond, VA 23219 USA; 2 University of Utah, Salt Lake City, UT USA

**Keywords:** Diabetes risk, Biomarker, Insulin resistance, Glycemic control, Beta cell function

## Abstract

Blood-based biomarker testing of insulin resistance (IR) and beta cell dysfunction may identify diabetes risk earlier than current glycemia-based approaches. This retrospective cohort study assessed 1,687 US patients at risk for cardiovascular disease (CVD) under routine clinical care with a comprehensive panel of 19 biomarkers and derived factors related to IR, beta cell function, and glycemic control. The mean age was 53 ± 15, 42 % were male, and 25 % had glycemic indicators consistent with prediabetes. An additional 45 % of the patients who had normal glycemic indicators were identified with IR or beta cell abnormalities. After 5.3 months of median follow-up, significantly more patients had improved than worsened their glycemic status in the prediabetic category (35 vs. 9 %; *P* < 0.0001) and in the “high normal” category (HbA1c values of 5.5–5.6; 56 vs. 18 %, *p* < 0.0001). Biomarker testing can identify IR early, enable and inform treatment, and improve glycemic control in a high proportion of patients.

**Electronic supplementary material:**

The online version of this article (doi:10.1007/s12265-014-9577-1) contains supplementary material, which is available to authorized users.

## Introduction

The prevalence of diabetes has reached epidemic proportions, affecting over 366 million people worldwide and more than 25 million in the USA alone. If present trends continue, one in three individuals will meet the criteria for diabetes by 2030 [1–3]. Prediabetes currently affects more than 87 million US adults (38 %) and confers a lifetime risk of conversion to diabetes of 30–50 % [4, 5]. Insulin resistance syndromes (diabetes, prediabetes, and metabolic syndrome) are associated with up to 70 % of cardiovascular disease (CVD) cases, and adults with diabetes are twice as likely to die from heart disease and stroke than those without diabetes [3, 6, 7]. The American Diabetes Association (ADA) estimates the cost of managing diabetes for just 1 year to average $7,900 per patient. If current trends continue, type 2 diabetes mellitus (T2DM) is projected to cost the USA $500 billion per year by 2020 [8, 9].

While by current practice diabetes is generally managed on the basis of fasting glucose and HbA1c [10–12], the pathophysiology-based view of prediabetes and diabetes is anchored in the paradigm that the root cause is insulin resistance in peripheral tissue, which triggers an initial, compensatory hypersynthesis of insulin by pancreatic beta cells [13–16]. Later-stage disease is characterized by pancreatic beta cell dysfunction, failure, and burnout, leading to relative—and in some cases absolute—hypoinsulinemia. When this occurs, circulating levels of insulin are not sufficient to overcome peripheral tissue insulin resistance, leading to dysglycemia and hyperglycemia. Furthermore, high insulin levels trigger the overproduction of very low-density lipoprotein (VLDL) particles in the liver, which, coupled with abnormal remodeling of these triglyceride-rich VLDL particles, leads to the development of “atherogenic dyslipidemia,” a triad of elevated triglycerides, high levels of small, dense LDL particles, and low levels of high-density lipoprotein cholesterol (HDL-C). Hyperglycemia coupled with atherogenic dyslipidemia leads to end-organ damage including atherosclerosis, neuropathy, nephropathy, and retinopathy [17].

It is now possible to assess insulin resistance [18–34], beta cell function [35–41], and glycemic control [10–12, 42–44] using peripheral blood-based biomarkers [45–47]. Accordingly, we hypothesized that a higher proportion of at-risk patients would be identified on the basis of comprehensive biomarker testing for insulin resistance and beta cell function compared to fasting glucose and HbA1c alone. Furthermore, we hypothesized that providers could intervene more aggressively based on these results and that patients would show improved glycemic control, hence, shift to a lower diabetes risk category when assessed at follow-up.

## Methods

### Study Design

This was a retrospective cohort study of 1,687 consecutive patients presenting for risk assessment and risk reduction at six prevention-focused outpatient clinics across the USA (Madison, WI; Jackson, MS; Montgomery, AL; Charleston, SC; Seattle, WA; and Salt Lake City, UT) enrolled between Apr 1, 2012 and May 27, 2013. Laboratory results were provided directly to each patient and physician and presented in a format designed to highlight abnormal values and engage patients, along with evidence-based treatment considerations relevant to each individual’s identified risk. The manner and degree to which biomarker data was used to guide treatment decisions was based solely on the discretion of each physician; no protocol-defined treatments were required. Family and medical history, current medications, vital signs, and demographic information were collected from chart review and matched to laboratory biomarker data, which were then de-identified. The study protocol was approved, and a waiver of informed consent granted by the Copernicus Group Institutional Review Board (IRB; Durham, NC) and University of Utah IRB.

### Laboratory Measurements

Comprehensive laboratory testing included 19 blood-based biomarkers and derived factors organized into three functional categories: (1) glycemic control, (2) insulin resistance, and (3) pancreatic beta cell function (Table 1). Fasting glucose was measured by an ultraviolet (UV) method; HbA1c by high-performance liquid chromatography; fructosamine by a colorimetric method; alpha-hydroxybutyrate (α-HB), oleic acid, and linoleoylglycerophosphocholine (L-GPC) by electrospray ionization LC-mass spectrometry; leptin, proinsulin, and anti-glutamic acid decarboxylase (anti-GAD) antibody by enzyme-linked immunosorbent assay; adiponectin by latex turbidimetric immunoassay; free fatty acids by an enzymatic colorimetric method and ferritin by a sandwich principle method; and insulin and C-peptide by electrochemiluminescence immunoassay. The glycation gap was calculated as: measured HbA1c − predicted HbA1c (0.01908 × fructosamine [μmol/L] + 1.099), and calculation of the “insulin resistance score” (IRi score) was adapted from [28]. The leptin/BMI and proinsulin/C-peptide ratios were calculated as leptin (ng/mL)/BMI and proinsulin (pmol/L)/C-peptide (ng/mL), respectively. The homeostatic model assessment of insulin resistance (HOMA-IR), a surrogate measure of insulin resistance, was calculated as: glucose (mg/dL) × insulin/405 (μU/mL). Reference ranges for “high range” of each biomarker were defined on the basis of cut points reported in the literature and based on internal analyses of population distributions derived from HDL, Inc. data (Table 1).

**Table 1:** Comprehensive biomarker panel for assessing glycemic control, insulin resistance, and beta cell function

Biomarkers	Biological function and/or clinical utility	High range
Glycemic control		
Glucose (mg/dL)	Fasting indicator of glucose homeostasis	>125
HbA1c (%)	Intermediate-term glycemic control (2–3 months)	≥6.5
Fructosamine (μmol/L)	Short-term glycemic control (2–3 weeks)	>339
Glycation Gap	Indicator of increased risk of glycemic tissue injury	>0.77
Insulin resistance		
α-Hydroxybutyrate (μg/mL)	Metabolomic marker of insulin resistance	>5.7
Oleic acid (μg/mL)	Metabolomic marker of insulin resistance	>79
Linoleoyl-GPC (μg/mL)	Metabolomic marker of insulin resistance	<10.5
IRi Score	Composite index calculated from metabolomic markers and fasting insulin level	<8
Leptin (ng/mL)	Adipokine regulates appetite and energy balance and links obesity with insulin resistance	>43
Leptin/BMI ratio	Marker of leptin resistance	>1.17
Adiponectin (μg/mL)	Adipokine—anti-inflammatory, stimulates beta oxidation of free fatty acids and enhances insulin sensitivity	<10
Free fatty acids (mmol/L)	Elevated in insulin resistance due to increased adipose tissue lipolysis and decreased beta oxidation may contribute to insulin resistance and vascular dysfunction	>0.7
Ferritin (ng/mL)	Iron transport protein and acute phase reactant elevated in association with insulin resistance and hemochromatosis	>108
HOMA-IR	Surrogate index of insulin resistance based on steady-state fasting insulin and glucose	>4.2
Beta Cell Function:		
Insulin (μU/mL)	Hyperinsulinemia	≥12
Proinsulin (pmol/L)	Hyperinsulinemia	>16
C-peptide (ng/mL)	Hyperinsulinemia	>4.6
Proinsulin/C-peptide ratio	Impaired insulin processing due to beta cell strain	>4.9
Anti-GAD antibody (IU/mL)	Indicator of islet autoimmunity occurs in type 1 diabetes or latent autoimmune diabetes of adults	>5

### Statistical Analysis

Patients were classified into glycemic categories (i.e., normal, prediabetic, and diabetic) using glucose and HbA1c levels according to the ADA diagnostic guidelines [10–12]. Importantly, these categories are not used here as diagnoses (as many patients were already taking anti-diabetic medications) but, rather, a means of assessing the level of glycemic control in a clinically meaningful way. Differences in patients’ clinical and demographic data among the glycemic categories were tested using one-way ANOVA and chi-squared tests for continuous and categorical data, respectively. The proportion of patients classified as prediabetic or diabetic according to ADA guidelines was compared to those identified with at least one IR or beta cell biomarker in the high range using the McNemar paired test. Age- and gender-adjusted linear models were used to test for linear trends in biomarker mean values among glycemic categories. The normoglycemic category was divided into “normal” and “high normal” as defined by fasting glucose <100 mg/dL but with HbA1c 5.5–5.6 % (consistent with guidelines from the American Association of Clinical Endocrinologists). Residual plots were inspected for normality and homoscedasticity, and biomarkers were transformed using the natural logarithm as needed to improve model assumptions. Multiple testing compared to the normoglycemic group was controlled using Dunnett adjusted *p* values <0.05 for statistical significance. The first visit during the study period was used for all cross-sectional analyses, and the first and last visits were used to analyze changes in glycemic categories. The proportions of patients in the high normal and prediabetic categories that changed glycemic categories during follow-up were tested using 2-proportion *Z* tests. All analyses were performed using StatView version 5 or SAS software (version 9.3; SAS Institute).

## Results

Patient characteristics at baseline by glycemic category (defined by fasting glucose and HbA1c) are shown in Table 2. A total of 1,687 patients were enrolled, mean (SD) age was 53 (15) years, and 704 (42 %) were male. Based on fasting glucose and HbA1c levels, 415 patients (25 %) had glycemic control consistent with prediabetes and 343 (20 %) with diabetes. These data reflect a high-risk cohort with almost half (48 %) meeting the current criteria for metabolic syndrome, which was also present in the normoglycemic group of patients (41 %). On average, the study population was obese, with almost one third and one half of the patients previously diagnosed with T2DM and/or hypertension, respectively. Those in the glycemic categories corresponding to prediabetes or diabetes were more likely to be obese, older males with elevated blood pressure and taking multiple pharmacotherapies. To note, 32 % of the patients classified as normoglycemic were receiving anti-diabetic treatment at the time of initial biomarker testing; presumably, these individuals had a medical history of hyperglycemia or metabolic syndrome (Table 2). Patients classified as diabetic had a significantly higher heart rate and stronger family history of both diabetes and heart disease. One in six patients (16 %) had been diagnosed with coronary artery disease (CAD). More than 60 % of patients were taking lipid-lowering medication (mostly statins), while nearly 50 % had been prescribed with anti-hypertensive and anti-inflammatory drugs.

**Table 2:** Patient baseline characteristics by glycemic category

	Number	All	Normal (55 %)	Prediabetic (25 %)	Diabetic (20 %)	*p* value^a^
Demographic						
Age (years)	1,687	53 (15)	50 (16)	57 (13)*	56 (12)*	<0.0001
Female	1,687	983 (58 %)	585 (63 %)	232 (56 %)*	166 (48 %)*	<0.0001
Non-Hispanic Caucasian	1,644	795 (48 %)	423 (47 %)	194 (48 %)	178 (53 %)	0.18
Clinical						
BMI (kg/m^2^)	1,618	30.2 (6.9)	28.7 (6.6)	31.1 (6.6)*	33.2 (7.0)*	<0.0001
Systolic BP (mmHg)	1,648	123 (17)	120 (16)	125 (17)*	127 (19)*	<0.0001
Diastolic BP (mmHg)	1,648	76 (10)	75 (10)	77 (11)*	77 (11)*	<0.0001
Heart rate (bpm)	1,633	74 (12)	73 (11)	74 (12)	77 (13)*	<0.0001
Currently smoking	1,589	81 (5.1 %)	41 (5 %)	22 (6 %)	18 (5 %)	0.82
Family history						
T2DM	1,236	507 (41 %)	258 (36 %)	111 (37 %)	138 (63 %)*	<0.0001
Hypertension	1,383	867 (62 %)	462 (59 %)	220 (64 %)	185 (71 %)*	0.0036
Premature CAD	1,193	371 (31 %)	186 (28 %)	92 (31 %)	93 (40 %)*	0.0021
Medical history						
T2DM	1,532	458 (30 %)	88 (10 %)	102 (26 %)*	268 (90 %)*	<0.0001
Hypertension	1,595	788 (49 %)	320 (36 %)	243 (62 %)*	225 (69 %)*	<0.0001
CAD	1,588	260 (16 %)	118 (14 %)	94 (24 %)*	48 (14 %)	<0.0001
Premature CAD	1,540	119 (7.7 %)	52 (6 %)	31 (8 %)	36 (11 %)*	0.017
Metabolic syndrome	1,530	733 (48 %)	335 (41 %)	219 (57 %)*	179 (54 %)*	<0.0001
Current medications						
Any anti-diabetic	1,686	777 (46 %)	297 (32 %)	172 (41 %)*	308 (90 %)*	<0.0001
Any lipid-lowering	1,682	1,089 (65 %)	533 (57 %)	292 (71 %)*	264 (77 %)*	<0.0001
Any anti-hypertensive	1,659	888 (54 %)	353 (39 %)	263 (64 %)*	272 (80 %)*	<0.0001
Any anti-inflammatory	1,659	730 (44 %)	361 (40 %)	204 (50 %)*	165 (48 %)*	0.0004

Data are mean (SD) or *n* (%) for continuous or categorical data, respectively

^a^One-way ANOVA and chi-squared test for continuous and categorical data, respectively

*p value < 0.05, multiple testing compared to the normal group was controlled using Dunnett method

Mean biomarker values for patients within each glycemic category are shown in Table 3. Significant linear trends indicating worsening abnormalities were observed for each biomarker in age- and gender-adjusted models across glycemic categories. Importantly, significant biomarker abnormalities (other than glycemic) were identified in the high normal glycemic group compared to the normal group, including leptin, adiponectin, linoleoyl-GPC, IRi score, HOMA-IR, insulin, and proinsulin.

**Table 3:** Comprehensive biomarker profiles by glycemic category, mean (SD)

*N* = 1,687	Normal (43 %)	High normal (12 %)	Prediabetic (25 %)	Diabetic (20 %)	Linear trend *p* value^a^
Glycemic control					
Glucose (mg/dL)	83 (9)	86 (8)*	97 (13)*	153 (59)*	<0.0001
HbA1c (%)	5.09 (0.24)	5.54 (0.05)*	5.73 (0.35)*	7.96 (1.56)*	<0.0001
Fructosamine (μmol/L)	224 (22)	225 (25)	227 (22)	303 (70)*	<0.0001
Glycation gap	−0.28 (0.47)	0.15 (0.47)*	0.31 (0.54)*	1.07 (1.05)*	<0.0001
Insulin resistance					
α-Hydroxybutyrate (μg/mL)	4.6 (2.4)	4.6 (2.1)	5.0 (2.4)*	6.4 (3.3)*	<0.0001
Oleic acid (μg/mL)	48 (26)	50 (24)	51 (27)	53 (29)*	0.016
Linoleoyl-GPC (μg/mL)	17.7 (6.5)	16.3 (6.1)*	16.0 (5.7)*	16.4 (6.3)*	<0.0001
IRi Score	12 (4)	11 (4)*	10 (4)*	11 (8)*	<0.0001
Leptin (ng/mL)	28 (28)	31 (29)*	39 (37)*	40 (37)*	<0.0001
Leptin/BMI ratio	0.90 (0.75)	0.97 (0.77)	1.15 (0.99)*	1.13 (0.96)*	<0.0001
Adiponectin (μg/mL)	18.5 (17.5)	17.7 (15.2)*	13.6 (9.3)*	11.1 (8.7)*	<0.0001
Free fatty acid (mmol/L)	0.52 (0.24)	0.55 (0.22)	0.55 (0.24)	0.57 (0.27)	0.050
Ferritin (ng/mL)	110 (107)	113 (122)	128 (167)	167 (165)*	<0.0001
HOMA-IR	1.9 (1.5)	2.4 (1.6)*	3.6 (3.2)*	7.8 (12.2)*	<0.0001
Beta cell function					
Insulin (μU/mL)	9.4 (6.9)	11.2 (7.5)*	15.0 (12.0)*	20.5 (27.3)*	<0.0001
Proinsulin (pmol/L)	10.1 (9.4)	12.1 (11.3)*	19.5 (17.2)*	30.8 (39.8)*	<0.0001
C-peptide (ng/mL)	2.5 (1.1)	2.9 (1.1)	3.5 (1.8)*	3.1 (2.4)*	<0.0001
Proinsulin/C-peptide ratio	4.0 (3.3)	4.1 (2.3)	5.6 (4.8)*	13.6 (11.8)*	<0.0001
Anti-GAD antibody positive: *n* (%)	23 (3.2)	6 (2.9)	15 (3.6)	53 (16)*	<0.0001

All biomarkers had <5 % missing data

^a^All continuous biomarkers were log transformed (except glycation gap) for improved normality and homoscedasticity of residual errors in age and gender adjusted linear models

*p value < 0.05, multiple testing compared to the normal group was controlled using Dunnett method

**Fig. 1:**
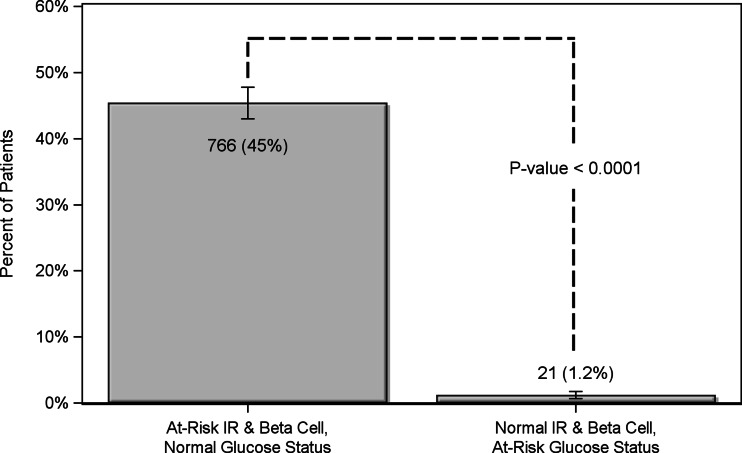
All patients were classified as at risk based on two criteria: first, high-range values of one or more biomarkers of insulin resistance (IR) or beta cell function, and second, traditional glycemic indicators (fasting glucose ≥ 100 mg/dL or HbA1c ≥ 5.7 %). The proportion of patients identified with one or more biomarkers of IR or beta cell function in the high range but normal glycemic indicators (glucose < 100 mg/dL and HbA1c < 5.7, *N* = 766) compared to the proportion of patients with abnormal glycemic but normal IR and beta cell function markers (*N* = 21) are shown. The two methods were in agreement for the remaining 900 patients. *N* = 1687, McNemar paired test *p* < 0.0001

There were 766 patients (45 %) classified with one or more biomarkers of IR or beta cell dysfunction in the high range that were not classified “at risk” by glucose or HbA1c. Conversely, 21 patients (1.2 %) were classified as prediabetic/diabetic on the basis of fasting glucose or HbA1c that had normal levels of IR and beta cell biomarkers (Fig. 1). The two methods were in agreement for the remaining 900 patients (*N* = 1687 McNemar paired test *p* value <0.0001). In order to investigate the possible effects of multiple testing, the random binomial (15, 0.256) and Poisson (3.8) distributions were compared to the observed distribution of positive tests, and the Kolmogorov–Smirnov statistical test was conducted to determine that the random distributions were significantly different than the observed distribution (*p* < 0.0001; Supplemental Figure 1).

The proportion of patients identified with biomarker abnormalities specific to either insulin resistance or beta cell function is shown in Table 4 grouped by traditional glycemic category and current anti-diabetic medication status. In the overall cohort, 1,424 (84.5 %) had at least one feature of insulin resistance and 967 (57.3 %) had at least one feature of beta cell dysfunction on comprehensive testing, while 1,078 (63.9 %) had at least two features of insulin resistance and 562 (33.3 %) had at least two features of beta cell dysfunction. Importantly, of those that were classified as normoglycemic according to the traditional criteria and *not* taking anti-diabetic medications, 77.2 % showed evidence of insulin resistance and 36.4 % had beta cell dysfunction on the basis of comprehensive testing (Table 4). Furthermore, patients on anti-diabetic medications who were classified normoglycemic according to traditional criteria showed significantly higher rates of beta cell dysfunction than those not on treatment (*p* < 0.05), suggesting that the goal on treating HbA1c may still leave significant residual risk (Table 4).

**Table 4:** Percentage of patients having biomarkers of insulin resistance or beta cell function in the high range by glycemic category and anti-diabetic medication status

Glycemic category	Insulin resistance (%)			Beta cell dysfunction (%)		
	All	YES anti-diabetic medications	NO anti-diabetic medications	All	YES anti-diabetic medications	NO anti-diabetic medications
% of patients having one or more (1+) high risk biomarker levels						
Normal (*N* = 929, 55 %)	78.4	80.8	77.2	39.8	47.1*	36.4
Prediabetes (*N* = 415, 25 %)	90.8	89.0	92.2	68.7	69.2	68.3
Diabetes (*N* = 343, 20 %)	93.3	93.5	91.2	91.0	90.6	94.1
All	84.5	87.6*	81.7	57.3	69.2*	47.1
% of patients having two or more (2+) high risk biomarker levels						
Normal (*N* = 929, 55 %)	54.7	58.9	52.7	16.0	19.5*	14.4
Prediabetes (*N* = 415, 25 %)	69.9	72.1	68.3	43.6	44.2	43.2
Diabetes (*N* = 343, 20 %)	81.6	81.2	85.3	67.4	66.6	73.5
All	63.9	70.7*	58.1	33.3	43.6*	24.3

*p value < 0.05, chi-squared test between patients using and not using anti-diabetic medications by glycemic category and overall

**Fig. 2:**
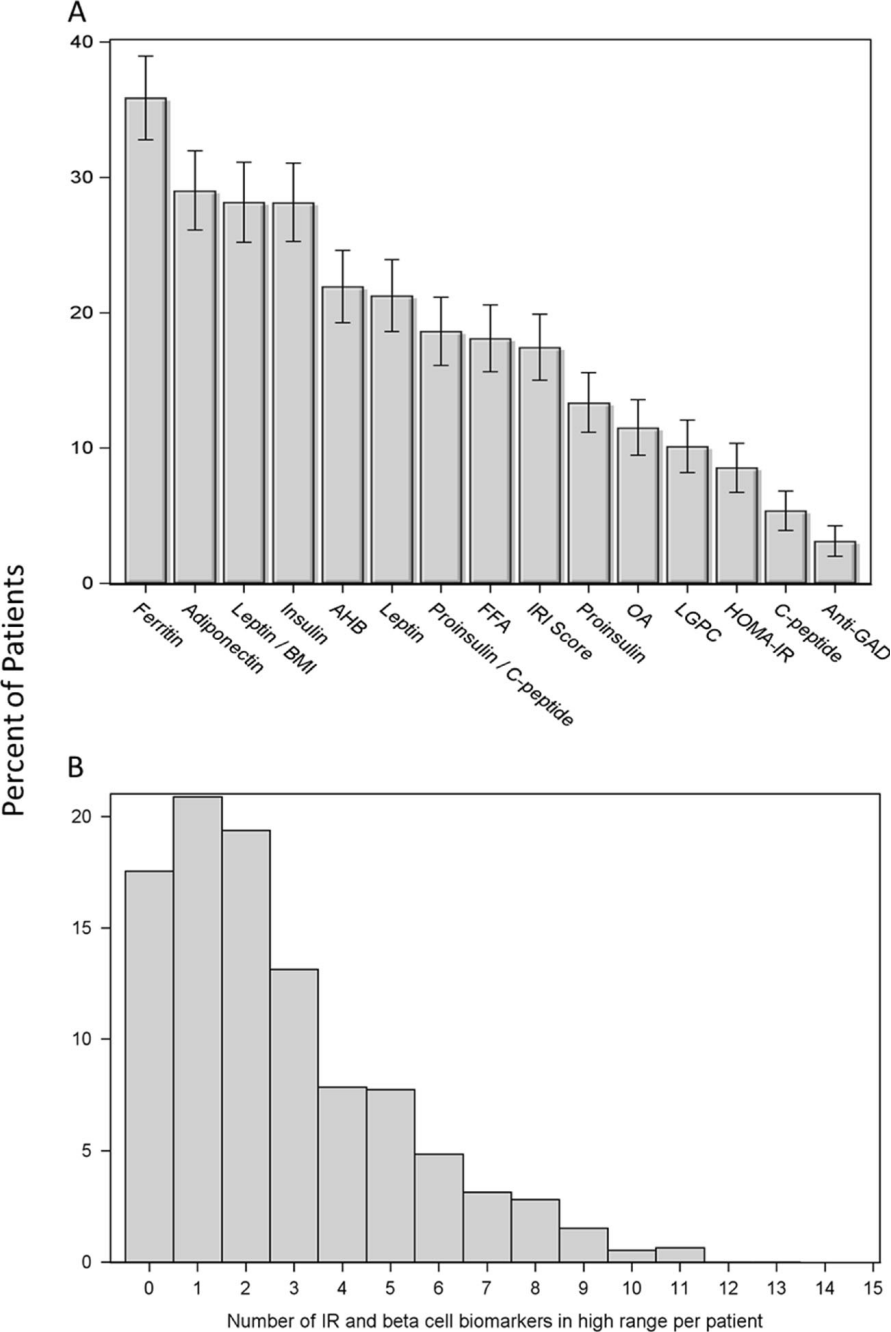
Evidence of insulin resistance and beta cell dysfunction in the normoglycemic patients is highly prevalent and heterogenous. Of those patients classified as normoglycemic (glucose < 100 mg/dL and HbA1c < 5.7, *N* = 929), 82 % demonstrated at least one high range biomarker of insulin resistance or beta cell function. **a** Proportion of normoglycemic patients demonstrating high range values of each biomarker; 95 % confidence limits are shown. **b** Distribution of the total number of high range biomarker values observed in normoglycemic patients

Figure 2 illustrates the degree and heterogeneity of underlying insulin resistance and beta cell dysfunction in the “normoglycemic” patients by showing which individual biomarkers were identified above the high range cut point. Of the 929 patients that were classified as normoglycemic on the basis of fasting glucose and HbA1c, 766 (82 %) showed at least one biomarker in the high range. As shown in Fig. 2a, there was a broad distribution of which biomarkers were elevated, with no single marker accounting for even half of this number. There were also differences noted in the number of biomarker abnormalities identified (Fig. 2b). Examination of individual patient biomarker profiles revealed that while there is often overlap between elevated markers, distinct patterns exist that would not be evident with a single biomarker or even a smaller combination of biomarkers (data not shown).

**Fig. 3:**
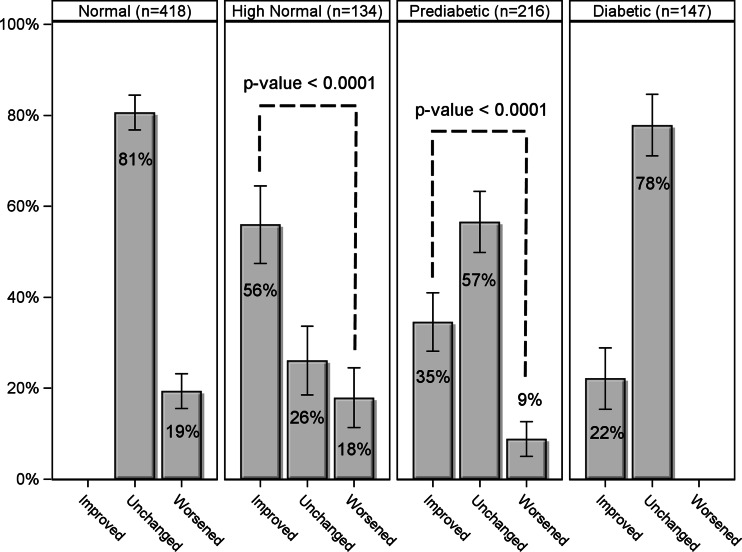
Changes in glycemic category associated with care guided by comprehensive biomarker testing. Patients were categorized based on glucose and HbA1c values obtained at the initial visit as either normal (glucose < 100 mg/dL and HbA1c < 5.5, *N* = 418), high normal (glucose <100 mg/dL and HbA1c 5.5–5.6, *N* = 134), prediabetic (glucose 100–124 mg/dL or HbA1c 5.7–6.4, *N* = 216), or diabetic (glucose ≥125 mg/dL or HbA1c ≥6.5, *N* = 147). The proportion of patients within each category who improved (by at least one category), remained unchanged, or worsened (by at least one category) upon retest (the median follow-up was 5.3 months) are shown. Patients in the prediabetic and high normal categories were three to four times as likely to improve than worsened (*p* value <0.0001); 95 % confidence limits are shown

A subset of patients (*N* = 915) had multiple biomarker panels performed during the study period, with a median interval of 5.3 months between the first and last tests. Patients that had follow-up visits were more likely to be non-white males with coronary artery disease; metabolic syndrome; and taking anti-diabetic, lipid-lowering, and anti-inflammatory medications; they also entered the study about 1 month earlier than those without a follow-up visit (all *p* < 0.05). At follow-up, a significantly higher proportion of those initially identified as prediabetic reverted to normoglycemic category than progressing to diabetes (35 vs. 9 %, *p* < 0.0001; Fig. 3), and a significantly higher proportion of those identified as high normal reverted to the normal category than worsened (56 vs. 18 %, *p* < 0.0001; Fig. 3). Overall, a significantly higher proportion of patients improved glycemic category rather than worsened despite the fact that the higher number of normal at baseline provided more opportunity for worsening (20 vs. 14 %, *p* = 0.0003). After grouping the patient data in Fig. 3 by patient medication status, a statistical test for homogeneity across medication status showed no significant differences in the percent change in glucose status between visits (*p* = 0.55).

## Discussion

This study evaluated the clinical utility of incorporating a comprehensive, multimarker panel into routine clinical care of patients at risk for, or with, diabetes. The results demonstrate three main findings. First, metabolic abnormalities were identified in a substantial proportion of patients who would not have been identified as prediabetic according to conventional glycemic cut points, thus providing opportunities for earlier intervention. Second, no single biomarker was responsible for this increased sensitivity—diverse patterns of biomarker abnormalities were observed—particularly in the normoglycemic patients. Finally, the improvements in glycemic category observed in the subset of patients for which follow-up data was available suggest that this multimarker approach can be successfully implemented in routine clinical practice to improve traditional goal attainment and, potentially, patient outcomes.

The ability to detect the risk for progression to diabetes earlier than is currently possible with the tools available to most clinicians is of critical importance in the effort to address the growing diabetes epidemic [5, 48, 49]. Earlier detection allows appropriate interventions to be administered when they are likely to be most effective. Preservation of pancreatic beta cell function has been shown to be the key to achieving good clinical outcomes [5]. Unfortunately, by the time dysglycemia is evident and a patient meets current criteria for prediabetes, substantial beta cell destruction has already occurred [5]. Since it is now well known that the vast majority of T2DM-associated beta cell destruction is preceded (by several years) by insulin resistance and increased compensating beta cell activity, identification and reversal of such abnormalities before damage occurs should be a goal of treatment. In the present study, 40 % of the cohort demonstrated signs of abnormalities related to insulin resistance and/or beta cell strain but had not yet lost glycemic control sufficient to meet traditional criteria for prediabetes. This represents a substantial increase in sensitivity of diabetes risk detection and a significant opportunity for early clinical intervention.

Much of this increased sensitivity results from the multimarker approach utilized in the present study. It is not surprising that as the focus of metabolic risk detection shifts to an earlier stage of the disease process, no single biomarker is able to identify every individual at risk. Insulin resistance and beta cell strain/dysfunction are not homogenous pathologies but, rather, are the result of multifactorial interactions between a large number of genetic and lifestyle risk factors. The development of prediabetes is usually identified first as either impaired fasting glucose (IFG) or impaired glucose tolerance (IGT), each presenting different diagnostic challenges and implications for progression of disease. Differences between these two states are often thought to reflect tissue specificity of insulin resistance; the etiology of which is most likely different in skeletal muscle, adipose tissue, and liver, with different biomarker signatures [50]. Other less frequent causes of diabetes such as autoimmune destruction of beta cells (e.g., late-onset autoimmune diabetes of adulthood) are often missed in their early stages but can easily be identified by assaying anti-GAD antibodies [41].

Conceptually, there are three main benefits to the multimarker approach in identifying diabetes risk. First, as described above, a panel of biomarkers provides increased sensitivity for detecting a heterogenous set of underlying pathological processes. Second, the number of abnormalities can be used in a practical sense to help stratify aggressiveness of intervention. For example, a borderline HbA1c value may be less worrisome if there is little evidence of insulin resistance, whereas even a normal HbA1c may be of concern if several aspects of insulin resistance and beta-cell function indicate risk. Finally, multimarker panels allow clinicians to learn about the unique pathobiology of individual patients which may help in personalizing care, engaging patients, and choosing the most effective treatments to slow or reverse the disease process [5, 18–21, 35]. While not designed as an outcomes study, the subset of patients in this analysis that had follow-up panels performed during the study period allowed the assessment of changes in glycemic categories. The observation that more than a third of patients categorized as prediabetic at first assessment had achieved normal glycemic control within 6 months suggests a significant improvement in their clinical care. This is particularly impressive considering that many of these patients had previously been diagnosed with diabetes and/or were already receiving anti-diabetic medications (as opposed to a cohort of newly identified prediabetics). As demonstrated in the Diabetes Prevention Program Outcomes Study (DPPOS), prediabetic patients who achieved even transient normal glycemic control during a 3-year intervention had their risk of diabetes reduced by more than 50 % over the subsequent 6 years [49]. Furthermore, the fact that more than half of the patients initially identified as high normal (i.e., HbA1c values 5.5–5.6) demonstrated improvements upon retest suggests that successful preventative measures were employed. It is important to note that no standardized intervention was employed in the patients described here, so conclusions are limited. However, the fact that these biomarkers were successfully integrated into routine practice is provocative.

The development of more effective treatment algorithms based on rapidly obtained and relatively inexpensive biomarker profiles that reflect underlying pathology is an important priority for both clinicians and researchers. One study demonstrating the effectiveness of “targeted pathophysiologic” therapy to reverse prediabetes was recently described [48]. High-risk patients were screened with an oral glucose tolerance test (OGTT), which was used to derive indices of both insulin resistance and beta cell function. A treatment algorithm based on the severity of these abnormalities was used to direct treatment; impressively, more than 50 % of prediabetic patients reverted to normal glucose tolerance. While logistical requirements of the OGTT limit its usefulness in most clinical settings, a panel of fasting blood markers may provide a practical alternative. Additional studies are underway to better understand the treatment implications of different biomarker profiles.

## Conclusions

In this study, we demonstrated the benefits of comprehensive, multimarker testing for insulin resistance and beta cell function to detect metabolic disease early on the insulin resistance/glycemia continuum, as a means to guide personalized dietary, lifestyle, and pharmacotherapy treatment regimens, and prevent and/or reverse the disease process. By using a comprehensive biomarker panel, we were able not only to appraise the severity of dysglycemia beyond traditional measures but also to identify physiological signs of insulin resistance in about 80 % of high-risk individuals who would have been classified as “normoglycemic” by conventional glycemic criteria. This clearly demonstrates the large number of individuals with normal glucose and HbA1c values who can be identified as having metabolic disease using a comprehensive biomarker panel. Prompt intervention in such patients may reverse the disease course and prevent progression to diabetes; this hypothesis will need to be tested in large, randomized, prospective clinical studies.

## Electronic supplementary material

Below is the link to the electronic supplementary material.

Supplemental Fig 1 The distribution for the number of observed positive tests is shown above for the entire population (N=1687); and the 15 IR and beta cell biomarkers had an overall 25.6% probability of reporting a positive test. If these positive tests were by chance, then the distribution could follow a Binomial distribution with n=15 and p=0.256, or a Poisson distribution with an average 3.8 positive tests per patient. Both of these random distributions have been overlaid with the observed distribution of positive tests, and the Kolmogorov–Smirnov statistical test was conducted to determine that the random distributions were significantly different than the observed distribution (p<0.0001). Specifically there were more patients with 0 or 1 positive test, fewer patients with 3 to 6 positive test results and more with over 6 positive tests than by chance; hence the observed distribution was not random. (PNG 54 kb)
