# Supplementary figures and images for: Comprehensive Biomarker Testing of Glycemia, Insulin Resistance, and Beta Cell Function Has Greater Sensitivity to Detect Diabetes Risk Than Fasting Glucose and HbA1c and Is Associated with Improved Glycemic Control in Clinical Practice
Source: J Cardiovasc Transl Res. 2014 Jul 29;7(6):597–606. doi: 10.1007/s12265-014-9577-1 (PMC4137169; doi:10.1007/s12265-014-9577-1)

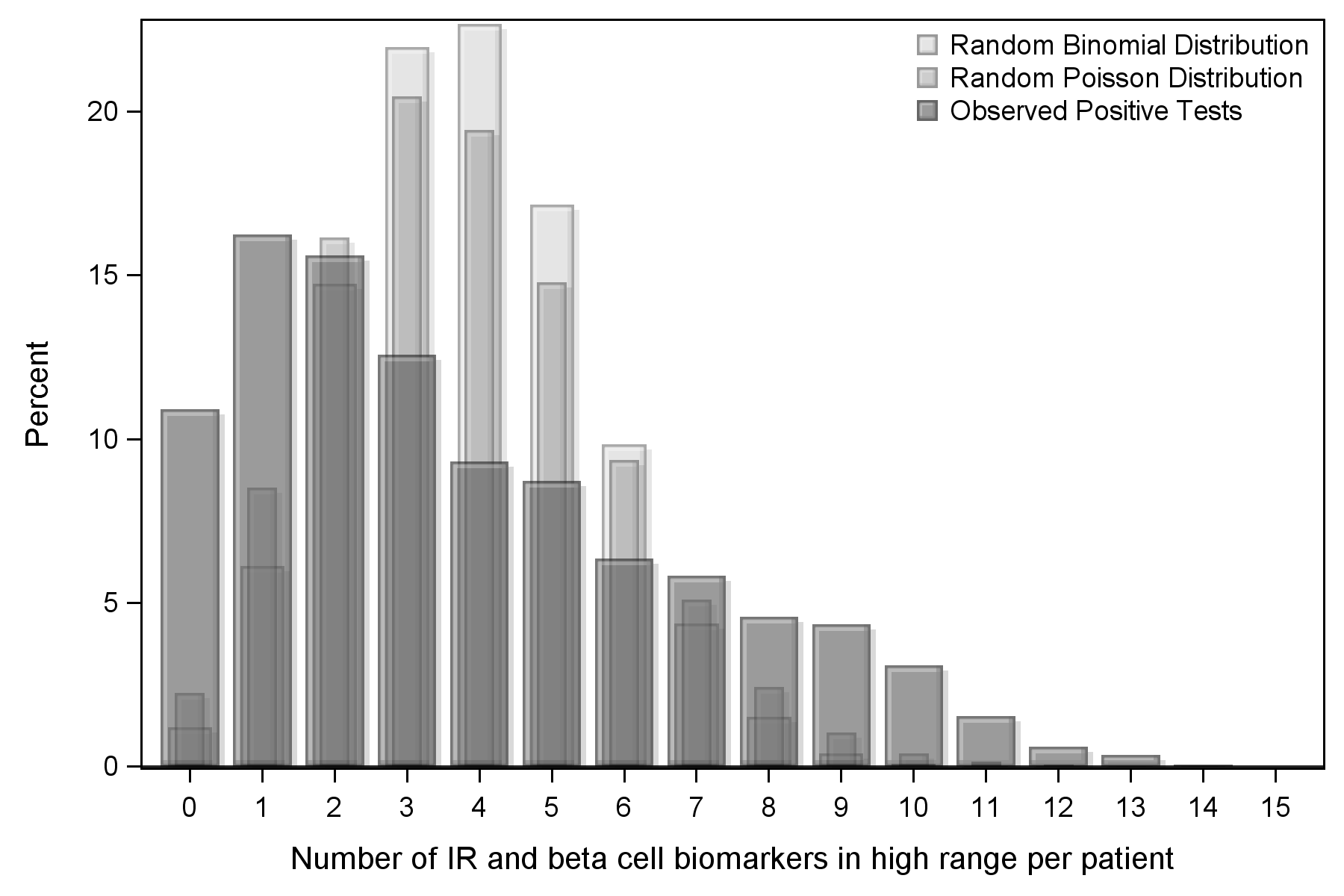

Supplement: Supplementary file 1 — The distribution for the number of observed positive tests is shown above for the entire population (N=1687); and the 15 IR and beta cell biomarkers had an overall 25.6% probability of reporting a positive test. If these positive tests were by chance, then the distribution could follow a Binomial distribution with n=15 and p=0.256, or a Poisson distribution with an average 3.8 positive tests per patient. Both of these random distributions have been overlaid with the observed distribution of positive tests, and the Kolmogorov–Smirnov statistical test was conducted to determine that the random distributions were significantly different than the observed distribution (p<0.0001). Specifically there were more patients with 0 or 1 positive test, fewer patients with 3 to 6 positive test results and more with over 6 positive tests than by chance; hence the observed distribution was not random. (PNG 54 kb) [file 12265_2014_9577_MOESM1_ESM.png]
